# Asymptomatic Transmissibility Calls for Implementing a Zero-COVID Strategy to End the Current Global Crisis

**DOI:** 10.3389/fcimb.2022.836409

**Published:** 2022-04-19

**Authors:** Chaobao Zhang, Hongzhi Wang, Zilu Wen, Mingjun Gu, Lianyong Liu, Xiangqi Li

**Affiliations:** ^1^ Department of Geriatric Medicine, Huadong Hospital, Shanghai Medical College, Fudan University, Shanghai, China; ^2^ Shanghai Key Laboratory of Clinical Geriatric Medicine, Huadong Hospital, Shanghai, China; ^3^ Shanghai Institute of Biochemistry and Cell Biology, Chinese Academy of Sciences, University of Chinese Academy of Sciences, Shanghai, China; ^4^ Shanghai Key Laboratory of Magnetic Resonance, East China Normal University, Shanghai, China; ^5^ Department of Scientific Research, Shanghai Public Health Clinical Center, Shanghai, China; ^6^ Department of Endocrinology, Gongli Hospital, The Second Military Medical University, Shanghai, China; ^7^ Department of Endocrinology, Punan Hospital, Shanghai, China

**Keywords:** COVID-19, SARS-CoV-2, asymptomatic spread, zero-COVID strategy, containment measure, software, artificial intelligence

## Abstract

The coronavirus disease 2019 (COVID-19) pandemic has led to unprecedented global challenges. A zero-COVID strategy is needed to end the crisis, but there is a lack of biological evidence. In the present study, we collected available data on SARS, MERS, and COVID-19 to perform a comprehensive comparative analysis and visualization. The study results revealed that the fatality rate of COVID-19 is low, whereas its death toll is high compared to SARS and MERS. Moreover, COVID-19 had a higher asymptomatic rate. In particular, COVID-19 exhibited unique asymptomatic transmissibility. Further, we developed a foolproof operating software in Python language to simulate COVID-19 spread in Wuhan, showing that the cumulative cases of existing asymptomatic spread would be over 100 times higher than that of only symptomatic spread. This confirmed the essential role of asymptomatic transmissibility in the uncontrolled global spread of COVID-19, which enables the necessity of implementing the zero-COVID policy. In conclusion, we revealed the triggering role of the asymptomatic transmissibility of COVID-19 in this unprecedented global crisis, which offers support to the zero-COVID strategy against the recurring COVID-19 spread.

## Highlights

- Asymptomatic spread is the fundamental difference between COVID-19 and SARS/MERS
- Our open-use software can simulate the asymptomatic spread
- Asymptomatic spread may be a trigger of the COVID-19 pandemic
- Ending the current global crisis needs the implementation of a zero-COVID strategy

## Introduction

Since COVID-19 (Coronavirus disease 2019) was first reported to the World Health Organization (WHO) on December 31, 2019, over 400 million cases of COVID-19 have been officially reported to date. It caused millions of death and devastated the economy and society. Despite various measures executed by different countries (Bayham and Fenichel, 2020; Hellewell et al., 2020; Koo et al., 2020; Mandal et al., 2020; Verelst et al., 2020; Zhang et al., 2020), the incidence of COVID-19 continues to surge worldwide. SARS (Severe acute respiratory syndrome) and MERS (Middle east respiratory syndrome) were quickly under control, whereas COVID-19 is still spreading outside China and several other countries. Success in countries like China has piqued the interest of some scientists. These scientists have theorized this as a zero-COVID strategy to end this crisis (Normile, 2021), but special biological evidence is needed.

We know that SARS and MERS were both reported to cause asymptomatic infection (Chan et al., 2003; Grant et al., 2019). Asymptomatic cases of COVID-19 have also been recorded (Chan et al., 2020; Novel Coronavirus Pneumonia Emergency Response Epidemiology Team, 2020; Wang, D. et al., 2020). Previous research suggested that substantial undocumented infection facilitates the rapid dissemination of COVID-19 (Li, R. et al., 2020). It is certain that asymptomatic infections are hidden and deceptive. We presume that the undocumented asymptomatic cases of COVID-19 develop key new features that further lead to uncontrollable rapid dissemination, thus fulfilling the need for the zero-COVID strategy.

Here we first investigated the epidemiology, infectivity, and transmissibility in detail with a special focus on asymptomatic infection of COVID-19 compared with SARS and MERS. Further, we developed a foolproof operating software in python language based on a susceptible-exposed-infected-removed (SEIR) mathematical model to simulate the asymptomatic spread of COVID-19. Using this software, we confirmed the critical role of asymptomatic spread in this pandemic. Thus, using data analysis and spread simulation, we here provide the evidence needed for a zero-COVID strategy.

## Methods

### SARS-CoV Data Collection

We searched international (ScienceDirect, PubMed, CNKI, EMBASE databases) and WHO databases using the term “SARS” or “Severe acute respiratory syndrome,” encompassing articles published after 2002. The search process was conducted in January 2020. Further, we manually searched the reference lists of the included studies. The case reports that included fewer than 20 patients were excluded as they were considered as having insufficient representative information and patient numbers. The raw data and relevant key references are available in the supplementary information (**Supplementary S1**, **S2**). All important clinical investigations and findings were graphically manipulated using GraphPad Prism 7.

### MERS-CoV Data Collection

We conducted a literature search in EMBASE databases and PubMed for studies of laboratory-confirmed MERS-CoV infection using the terms: “MERS” or “MERS-CoV”, “asymptomatic” or “prevalence” and “seroprevalence” or “serological” or “infection”. Further studies were identified using the bibliography of the relatively recent published reviews and the WHO MERS technical network. Only articles in English published before March 1, 2020, were included. The case reports that included fewer than 20 patients were excluded as they had insufficient representative information and patient numbers. The raw data and relevant key references are available in the supplementary information (**Supplementary S1**, **S2**).

### SARS-CoV-2 Data Collection

In the early course of the SARS-CoV-2 outbreak, the term SARS-CoV-2 was not yet coined. Thus, we conducted a focused search to identify any missed relevant open reports and publications using various search terms, such as “2019-nCoV” or “Wuhan” or “Virus” in English and Chinese. After the SARS-CoV-2 was named by the International Committee on Taxonomy of Viruses (ICTV), we utilized the following search terms: “2019-nCoV” or “SARS-CoV-2” or “COVID-19”. We collected data using ScienceDirect, PubMed, CNKI, EMBASE, Baidu, and Google in English and Chinese.

We collected data on asymptomatic infection cases using the following search terms: “asymptomatic”, “asymptomatic infection”, “asymptomatic transmission”, “asymptomatic spread.” Since COVID-19 was first reported in Wuhan, China, we conducted a focused search for obtaining the early asymptomatic infection data in the official data of China (CDC or official media). In addition, all the included asymptomatic infection cases should be laboratory-confirmed (nucleic acid testing). Only articles or data on asymptomatic infections in English or Chinese were included. All the data before April 16, 2020, were considered. The routine data were primarily collected from WHO, and partial data were collected from the public reporting data (CDC or official media). The raw data and all relevant references are available in the supplementary information (**Supplementary S1**, **S2**).

### Developing the Open-Use Software and Simulating Asymptomatic Transmissibility

SEIR mathematical models are generally used to analyze the spread of infectious diseases. However, these SEIR models are presented in mathematical formulas, and non-professionals cannot interpret them. In a sense, the COVID-19 crisis offers a historic opportunity for the development of artificial intelligence in epidemiological research (Xiao et al., 2021). We previously adopted the SEIR model to simulate viral transmissibility and developed the K-SEIR software (Wang, H. et al., 2021). However, K-SEIR software could not simulate the asymptomatic spread. Here, we developed an improved version called SEIR-AS, thereby targeting the asymptomatic spread.

To put it simply, the SEIR model classified the population into the following six categories: Susceptible (S) with an average daily infection rate of λ; Exposed (E) with a parameter of the probability of a carrier turning into an infected case per day σ; Infectious (I); Removed (R). The average daily treatment probability for infected patients is γ, comprising two parameters: healing rate α and mortality rate β. The K-SEIR model added the artificial intervention parameter K. SEIR-AS model added the parameters of asymptomatic population and spread. Further, it added two key parameters, α and ρ, and simultaneously, the change healing rate α was presented as 1-β. Here, α indicates the proportion of asymptomatic cases in total cases, and ρ indicates the average daily infection rate of asymptomatic cases. Other specific computations are recalculated as needed. Software engineering was used to convert the theoretical SEIR-AS model into a basic PYTHON software. The software is offered for noncommercial use only and can be downloaded for free. The software and instructions are available online (http://peiyun.cn/download/seir_sim.files/SEIR-AS_en_V3.42.exe; http://www.peiyun.cn/news/software_seir_simulator_show.html).

## Results

### The Spread of COVID-19 Is Widespread and Persistent Around the World

**Figure 1:**
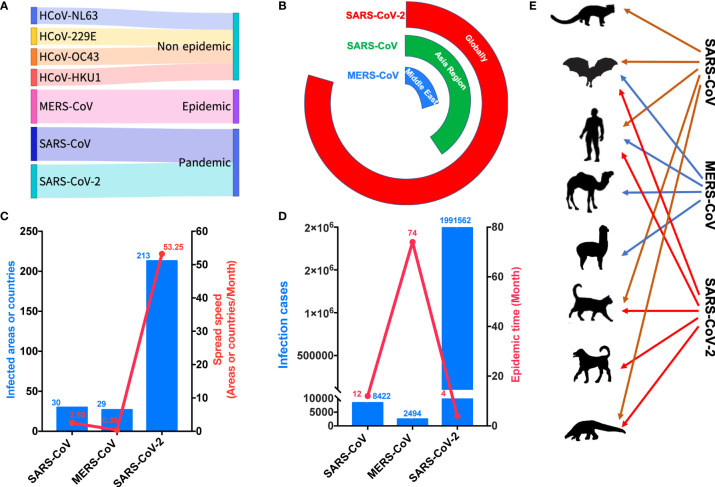
COVID-19 is creating the worst global pandemic. **(A)** Seven coronaviruses that can infect humans have led to different epidemic consequences. **(B)** SARS-CoV-2 sparked a global pandemic. **(C)** SARS-CoV-2 was associated with infection in the highest number of countries and exhibited the highest speed of transmission. **(D)** SARS-CoV-2 infected the largest number of people in the shortest time. **(E)** SARS-CoV-2 infected specific animals that come in close contact with humans. HCOV-NL63, human coronavirus NL63; HCOV-299E, human coronavirus 299E; HCOV-OC43, human coronavirus OC43; HCOV-HKU1, human coronavirus HKU1; MERS-CoV, Middle East respiratory syndrome coronavirus; SARS-CoV, Severe acute respiratory syndrome coronavirus. SARS-CoV-2, Severe acute respiratory syndrome coronavirus 2.

Seven coronaviruses that can infect humans have been identified. Four of them cause mild upper respiratory diseases and are not epidemic. Meanwhile, MERS-CoV caused an epidemic, whereas SARS-CoV and SARS-CoV-2 both created pandemics (**Figure 1A**). However, these viruses are dissimilar in many ways. In terms of their spread, SARS-CoV presented mainly in Asia, especially in China, whereas MERS-CoV was primarily localized to the Middle East, although it was also detected in South Korea. Conversely, SARS-CoV-2 has been detected in nearly all countries (**Figure 1B**). SARS-CoV spread to 30 countries at a speed of 2.5 countries per month, MERS-CoV spread to 27 countries at a speed of 0.36 countries per month, and SARS-CoV-2 spread to more than 200 countries at a speed of 53.25 countries per month (**Figure 1C**). More than 8000 persons experienced SARS-CoV over 12 months, whereas MERS-CoV was detected in more than 2000 persons over 74 months (**Figure 1D**). However, more than 170 million people have presented with SARS-CoV-2 within 4 months (**Figure 1D**). Interestingly, SARS-CoV has been detected mainly in humans, civets, cats, ferrets, and bats, versus humans, bats, dromedaries, and alpacas for MERS-CoV (**Figure 1E**). Contrarily, SARS-CoV-2 has been detected mainly in humans, cats, ferrets, dogs, bats, and pangolins (**Figure 1E**).

### The Fatality Rate of COVID-19 Is Low, but the Death Toll Is High

**Figure 2:**
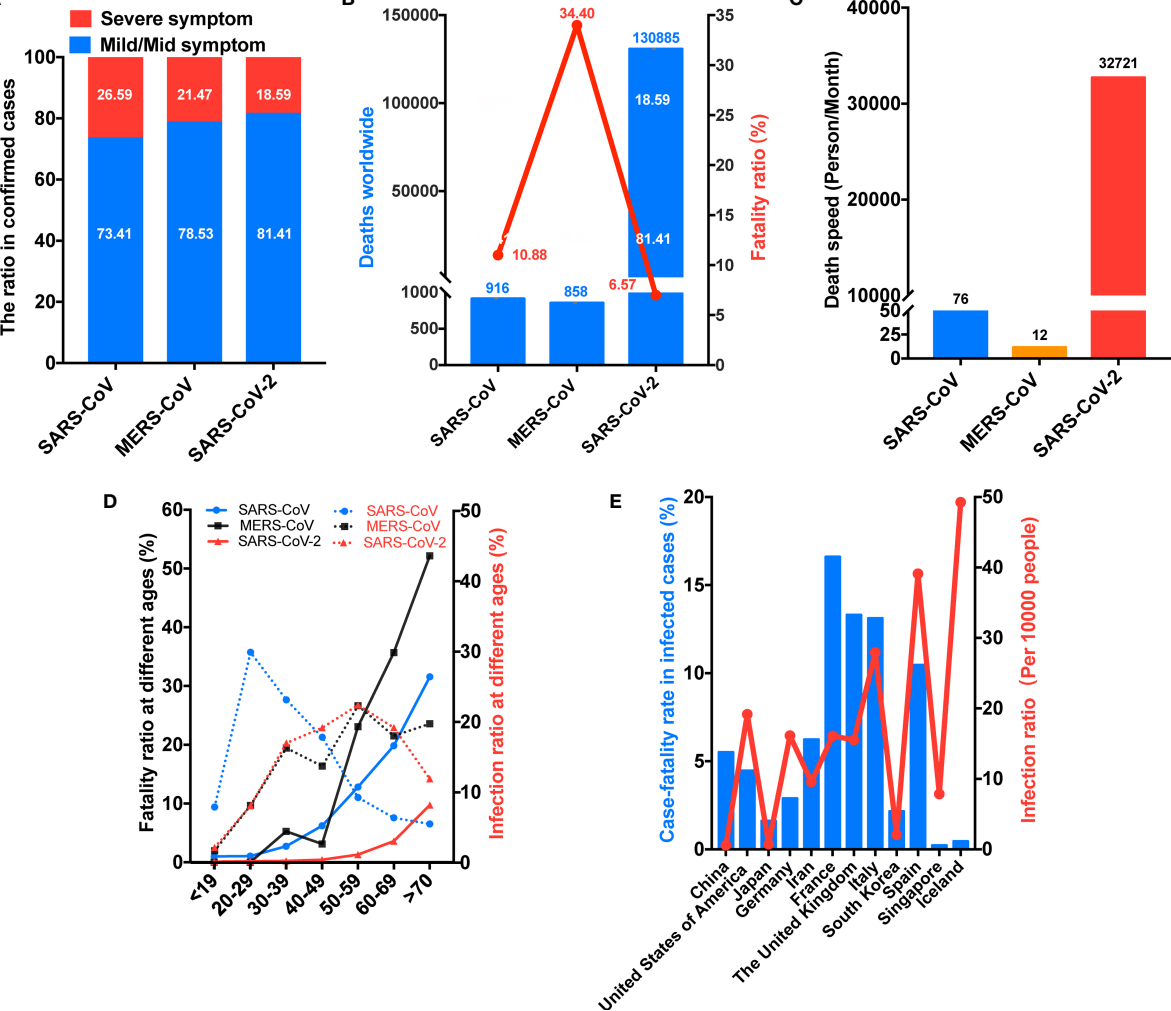
COVID-19 is leading to more deaths. **(A)** SARS-CoV-2 was linked to the highest rate of mild-to-moderate pneumonia. **(B)** SARS-CoV-2 has caused the highest number of deaths despite having the lowest mortality rate. **(C)** SARS-CoV-2 caused the highest number of deaths per month. **(D)** SARS-CoV-2 mainly infected middle-aged and elderly people, but the age-related increase in mortality was delayed. **(E)** The infection and mortality rates of SARS-CoV-2 varied by country. SARS-CoV, Severe acute respiratory syndrome coronavirus; MERS-CoV, Middle East respiratory syndrome coronavirus. SARS-CoV-2, Severe acute respiratory syndrome coronavirus 2.

In terms of disease severity, over 60% of SARS-CoV cases were associated with mild symptom, while this proportion for MERS-CoV and SARS-CoV-2 cases was over 70% and 80%, respectively (**Figure 2A**). SARS-CoV was linked to an overall mortality rate of 10.88% versus 34.40% for MERS-CoV (**Figure 2B**). Conversely, the mortality rate of SARS-CoV-2 has been 6.57% (**Figure 2B**). However, in terms of total deaths, SARS-CoV and MERS-CoV have been responsible for 916 and 858 deaths, while SARS-CoV-2 caused more than 13,000 deaths (**Figure 2B**). Concerning the numbers of deaths per month, SARS-CoV and MERS-CoV were linked to 76 and 12 deaths per month, respectively (**Figure 2C**). Unexpectedly, SARS-CoV-2 has been linked to an alarming 32,721 deaths per month. Meanwhile, infection and mortality rates have varied by age. In terms of infection rates, SARS-CoV had the highest infectivity in adults aged 20–29 years and the lowest in adults older than 70 years, whereas both MERS-CoV and SARS-CoV-2 had the highest infectivity in adults aged 50–59 years and the lowest in people younger than 19 years (**Figure 2D**). In terms of mortality rates, both SARS-CoV and MERS-CoV were linked to greater mortality in patients 30 years and older, but the increase of the mortality rate with increasing age was larger for MERS-CoV than for SARS-CoV (**Figure 2D**). Contrarily, substantial increases in mortality rates for SARS-CoV-2 only appear in patients older than 50 years (**Figure 2D**). The mortality rates of SARS-CoV-2 are lower than those of SARS-CoV and MERS-CoV at all ages (**Figure 2D**). In addition, infection and mortality rates have varied by country. For example, Iceland, Italy, and Spain have experienced infection rates exceeding 25 per 10,000 persons, and France, the UK, Italy, and Spain have reported mortality rates exceeding 10% (**Figure 2E**). Interestingly, China had an infection rate of less than 1 per 10,000 persons and a mortality rate of approximately 5%, whereas Iceland recorded an infection rate of approximately 50 per 10,000 persons and a mortality rate of less than 1% (**Figure 2E**).

### COVID-19 has the Highest Maximal Incubation Periods and Unique Asymptomatic Transmissibility

**Figure 3:**
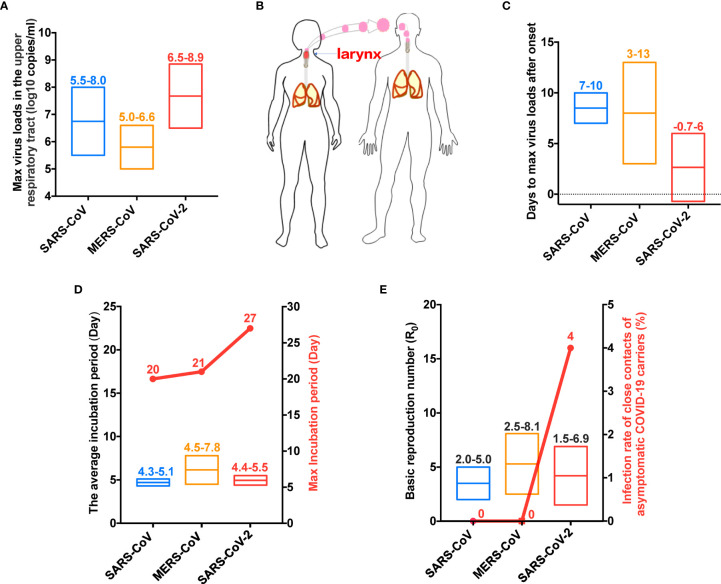
COVID-19 has specific virologic characteristics. **(A)** SARS-CoV-2 was associated with the highest viral load in the upper respiratory tract. **(B)** SARS-CoV-2 primarily replicated in the larynx in the early stage. **(C)** SARS-CoV-2 had the shortest time to peak viral load in the upper respiratory tract. **(D)** SARS-CoV-2 had the longest maximal incubation period. **(E)** SARS-CoV-2 had a high basic reproduction number (R0), but the key was the hidden transmission ability. SARS-CoV, Severe acute respiratory syndrome coronavirus; MERS-CoV, Middle East respiratory syndrome coronavirus. SARS-CoV-2, Severe acute respiratory syndrome coronavirus 2.

The maximal viral loads in the upper respiratory tract were approximately 5.5–8.0 (log10 copies/ml) for SARS-CoV, 5.0–6.6 for MERS-CoV, and 6.5–8.9 for SARS-CoV-2 (**Figure 3A**). SARS-CoV-2 mainly replicates in the larynx at the early stage of infection (**Figure 3B**). After symptom onset, the time to maximal viral loads was 7–10 days for SARS-CoV, 3–13 days for MERS-CoV, and −0.7–6 days for SARS-CoV-2 (**Figure 3C**). The average incubation periods for these three viruses were 4.3–5.1, 4.5–7.8, and 4.4–5.5 days, respectively (**Figure 3D**), whereas their maximal incubation periods were 20, 21, and 27 days, respectively (**Figure 3D**). The basic reproduction numbers (R0) of SARS-CoV, MERS-CoV, and SARS-CoV-2 were 2.0–5.0, 2.5–8.1, and 1.5–6.9, respectively (**Figure 3E**). Most remarkably, asymptomatic SARS-CoV and MERS-CoV carriers exhibited no infectious capacity, whereas the infection rate in the close contacts of asymptomatic SARS-CoV-2 carriers was 4% (**Figure 3E**).

### COVID-19 has a High Asymptomatic Rate for Confirmed Cases and the Contacts of Confirmed Cases

**Figure 4:**
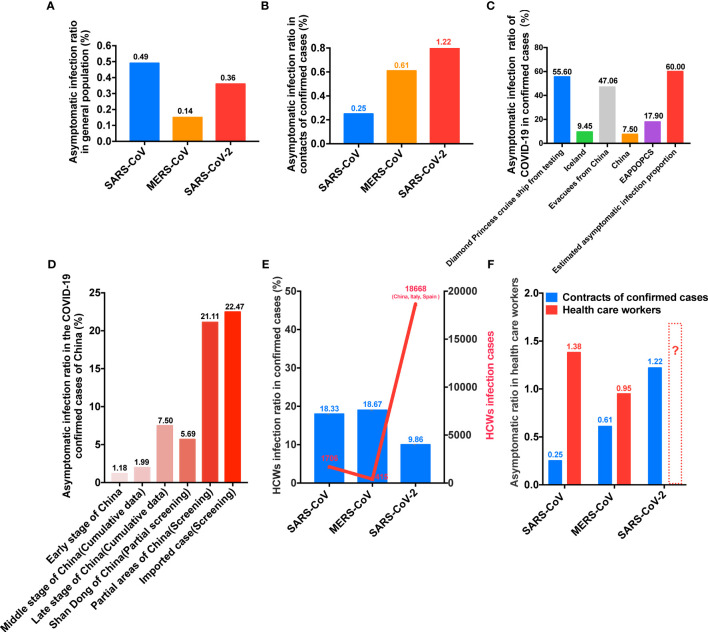
COVID-19 has unique asymptomatic spread. **(A)** The asymptomatic infection rate of SARS-CoV-2 in the general population was higher than those of SARS-CoV and MERS-CoV. **(B)** SARS-CoV-2 was associated with the highest asymptomatic infection rate among people exposed to confirmed cases. **(C)** SARS-CoV-2 was linked to high asymptomatic infection rates among confirmed cases from various samples (EAPDOPCS: estimated asymptomatic proportion on the Diamond Princess cruise ship from a model study). **(D)** Cumulative asymptomatic data of SARS-CoV-2 infection in China for different samples. **(E)** SARS-CoV-2 was linked to high numbers of infected HCWs. **(F)** HCWs had high rates of asymptomatic infection. SARS-CoV, Severe acute respiratory syndrome coronavirus; MERS-CoV, Middle East respiratory syndrome coronavirus. SARS-CoV-2, Severe acute respiratory syndrome coronavirus 2. HCWs, healthcare workers.

Concerning the asymptomatic infection rate in the general population, rates of 0.49, 0.14, and 0.36% have been recorded for SARS-CoV, MERS-CoV, and SARS-CoV-2, respectively (**Figure 4A**). Meanwhile, regarding the contacts of confirmed cases, the asymptomatic infection rates of these viruses were 0.25, 0.61, and 1.22%, respectively (**Figure 4B**). The asymptomatic rate for SARS-CoV-2 for passengers on the Diamond Princess cruise ship was 55.60%, and a rate of 47.06% has been reported for evacuees (Japan, South Korea, Germany, Singapore, and France) from China (**Figure 4C**). In addition, asymptomatic rates of 9.45 and 7.50% were observed in Iceland and China, respectively (**Figure 4C**). Meanwhile, it has been estimated that the proportion of patients from the Diamond Princess cruise ship with asymptomatic infection was 17.90% in a model study, and an additional estimate suggested that the total proportion of asymptomatic patients was 60.00% (**Figure 4C**). Among confirmed cases of SARS-CoV-2 infection, the total asymptomatic rates in the early, middle, and late stages of the pandemic in China (cumulative data) were 1.18, 1.99, and 7.50%, respectively (**Figure 4D**). In selected areas of China (screening), the rate increased to 21.11%, and the rate among imported cases was 22.47% (**Figure 4D**). We also assessed data for healthcare workers (HCWs). In this group, the infection rates for SARS-CoV, MERS-CoV, and SARS-CoV-2 were 18.33, 18.67, and 9.86%, respectively (**Figure 4E**). However, the total case number of HCWs with SARS, MERS and COVID-19 to date have been reported as 1706, 415, and 18,668, respectively (**Figure 4E**). For SARS-CoV, the asymptomatic rate for contacts of confirmed cases was 0.25% (3/1220), compared with 1.38% (49/3544) for HCWs (**Figure 4F**). For MERS-CoV, these values were 0.61 (8/2143) and approximately 0.95% (11/1157), respectively. For SARS-CoV-2, the asymptomatic infection rate for contacts of confirmed cases was 1.22% (61/4304), whereas the data for HCWs remain to be reported (**Figure 4F**). It is presumed that the rate will be higher than that recorded for the contacts of confirmed cases.

### Zero-COVID Strategy Plays a Crucial Role in Controlling Imported Asymptomatic Cases at the Late Stage of the COVID-19 Pandemic

**Figure 5:**
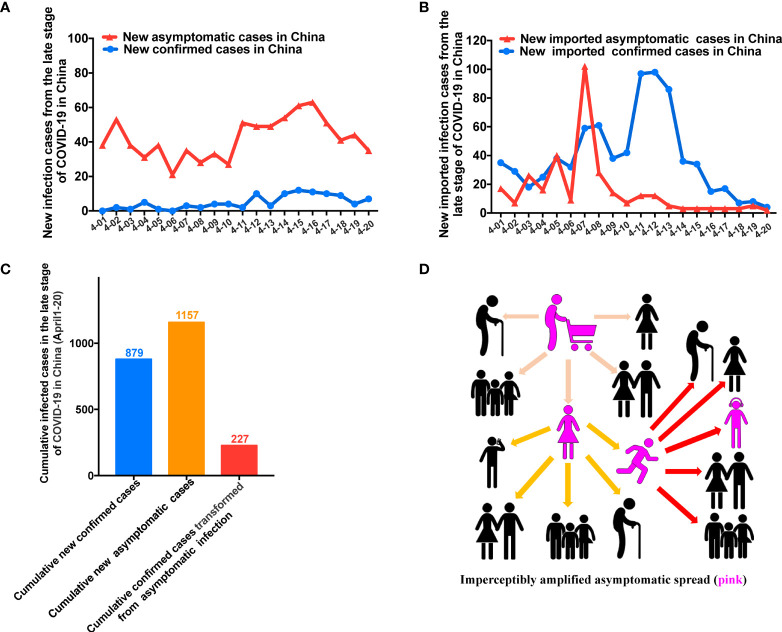
China curbed asymptomatic spread in its late stage of COVID-19. **(A)** Most cases of COVID-19 in the late phase of the pandemic in China were asymptomatic. **(B)** The rate of asymptomatic infection among imported cases in the late stage of the pandemic in China was relatively high. **(C)** Among the newly diagnosed cases, a higher proportion exhibited asymptomatic infection in the late phase of the pandemic in China than in the early phase. **(D)** Asymptomatic infection (pink) can lead to imperceptible subexponential or exponential transmission.

China recently entered the late stage of the SARS-CoV-2 pandemic. We analyzed cases of infection diagnosed in mainland China since April 1, 2020, and found that the majority of cases involved asymptomatic infection (**Figure 5A**). This trend was also observed among imported cases diagnosed since April 1, 2020 (**Figure 5B**). The accumulated data indicated that recently diagnosed asymptomatic infections outnumber recently diagnosed symptomatic infections (**Figure 5C**). Asymptomatic infections could be seeding new outbreaks. As such, they will lead to subexponential or exponential amplification, causing uncontrolled and undetected transmission (**Figure 5D**).

These data from the late stage of the pandemic in China illustrated the seriousness of asymptomatic transmission. At this late stage of the pandemic in China, the main task is preventing the spread of imported and asymptomatic infections to avoid a recurrent outbreak. For example, as per an open report, a recent asymptomatic case spread to more than 100 people in Jilin, China (People’s Daily, January 17, 2021, **Supplementary S3**). Thus, preventing asymptomatic spread is critical for halting this deadly disease. In response, China continued the zero-COVID strategy and successfully contained the epidemic without major outbreaks.

### Simulation of COVID-19 With Asymptomatic Transmission Fits the Real Scene but Does Not Fit With No Asymptomatic Transmission

**Figure 6:**
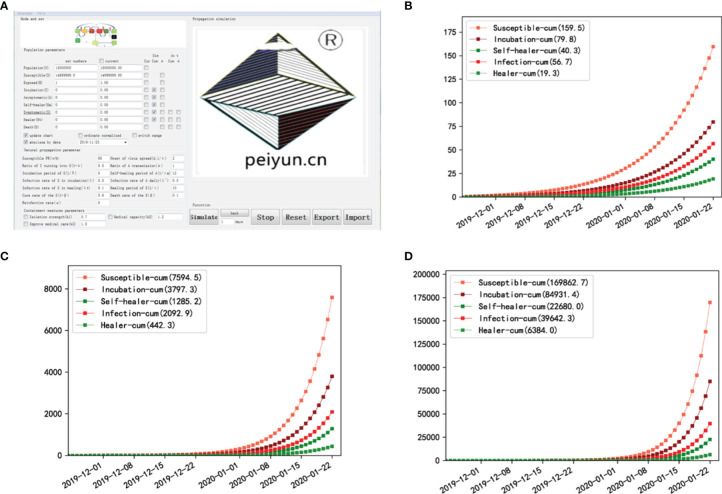
Simulation of asymptomatic transmissibility shows its triggering effect. **(A)** Interface diagram after software startup. **(B)** Simulation data in Wuhan, China show that cumulative cases are 56.7 when there is no asymptomatic spread. **(C)** Simulation data in Wuhan when one-half of asymptomatic infection is spread. There will only 2,092.9 cumulative cases. **(D)** Simulation data in Wuhan when all the asymptomatic infection is spread. There will be several 10,000 cases, which may be the case [the present time (39,642.3) vs. the final (approximately 50,000)].

To model viral transmissibility, we previously built the K-SEIR software. However, the K-SEIR program was unable to simulate asymptomatic spread. We built a new operational version dubbed SEIR-AS (**Supplementary Data**) based on a modified SEIR mathematical model to simulate SARS-CoV-2 spread in Wuhan and thus provide additional support for the crucial role of asymptomatic spread. SEIR-AS model added asymptomatic indexes. And it added two key parameters, α and ρ, to characterize the proportion of asymptomatic cases in total cases and the average daily rate of infection of asymptomatic cases, respectively. At the same time, the change healing rate α was presented as 1-β based on the K-SEIR model (**Figure 6A**). Other particular computations were adjusted accordingly. The theoretical model was translated into basic software implemented in the Python programming language using software engineering.

The software has two modes of operation, data-driven and model-driven, and the latter is used here. When we entered the SARS-CoV-2 epidemiological characteristics (**Figure 6A**), we discovered that if asymptomatic cases cannot spread, the cumulative number of cases at 60 days after the commencement of the disease by one carrier is 56.7 in a population of 15 million (**Figure 6B**). This is far from the truth. When one-half of asymptomatic infections can spread, 2092.9 cases will be presented (**Figure 6C**). This number of thousands of infections is still far fewer than the total number of infections. If all of the asymptomatic cases spread, the case number will be 39,642.3; this case number of several tens of thousands appears to be accurate (**Figure 6D**). Thus, asymptomatic transmission indeed plays a critical role in the outbreak of SARS-CoV-2.

## Discussion

The impact of COVID-19 on human beings has been so great that it has been called “WARS,” implying that it has the same impact as wars (Liu et al., 2021). But the underlying factor causing the uncontrollable spread remains to be uncovered. We here are figured out to reveal the root factor by comparing it with two other deadly viral infections, SARS and MERS. We found that the only fundamental difference between COVID-19 and SARS/MERS is the asymptomatic transmissibility of COVID-19 (**Figure 3E**) (Mizumoto et al., 2020). COVID-19 was linked to a high asymptomatic rate. The high asymptomatic rate has been primarily reflected in data for evacuees and special samples such as cruise ship passengers (**Figure 3D**). The asymptomatic rate of over 50% in the late stage of the pandemic in China confirmed the previous high estimate and fully illustrated the seriousness of asymptomatic transmission (**Figure 5**) (Qiu, 2020). Second, asymptomatic infection was previously ignored, possibly because asymptomatic patients with SARS and MERS do not transmit the virus (**Figure 3E**). Crucially, we uncovered that the asymptomatic spread of COVID-19 is too well hidden to see and was only confirmed by laboratory tests combined with epidemiological investigations (**Figure 3E**) (Hu et al., 2020; Li, C. et al., 2020). Asymptomatic infection caused most cases to be missed on screening (Gostic et al., 2020). Plus, asymptomatic patients have similar viral loads as symptomatic patients (Danis et al., 2020). Above all, we discovered that China is a good example of how to stop the asymptomatic spread.

China may have effectively controlled the outbreak by inadvertently blocking the transmission by segregating close contacts and encouraging mask use (Zhang et al., 2020). At the end of the outbreak, China’s emphasis on asymptomatic investigation demonstrates a growing knowledge of the importance of asymptomatic infection. Crucially, through software simulation, we confirmed the key role of asymptomatic transmission in expanding this pandemic (**Figure 6**). As a side note, this user-friendly software is available for free (**Supplementary Data**) to both professional scientists and individuals, institutions, and social organizations interested in epidemic analysis. These findings highlight the need to execute the COVID-19 zero-spread strategy by halting asymptomatic transmission.

Current research on asymptomatic symptoms is relatively lacking. The immune mechanisms of asymptomatic infection must be clarified. Because of the asymptomatic transmissibility of COVID-19 (**Figure 3E**) (Bai et al., 2020; Chan et al., 2020; Hoehl et al., 2020; Li, C. et al., 2020; Rothe et al., 2020), we must study the immune status of asymptomatic patients, predict the likelihood of large-scale infection, and analyze whether viruses can live in the body for a long time or exist symbiotically. A nonhuman primate model was recently developed to study the pathogenesis of COVID-19, providing a key foundation for studying the mechanisms of asymptomatic immunity (Rockx et al., 2020). Very proactively, a newly published clinical investigation uncovered the protective immunity in asymptomatic patients (Gao et al., 2021). Still, more investigations remain to be performed. The epidemic characteristics of asymptomatic transmission should also be investigated. Current clinical investigations of asymptomatic infection are sporadic (Hu et al., 2020; Li, C. et al., 2020; Lin et al., 2020; Rothe et al., 2020; Wang, Y. et al., 2020). A systematic and comprehensive investigation is required. Crucially, we must investigate the epidemic characteristics of the novel coronavirus in combination with global geographic, social, and climatic differences to confirm whether this novel coronavirus can achieve long-term coexistence with humans, which may be induced by a prolonged local epidemic associated with asymptotic transmission. Early warning and surveillance programs should additionally be developed.

To bring asymptomatic infections under control, we should formulate suitable containment measures. Because COVID-19 is a respiratory infectious disease, basic constraint measures such as disinfecting the public environment regularly, large-scale testing, effective self-isolation, social distancing, vaccination, and travel limitations should be employed by all countries, whereas other measures should be selected according to country-specific conditions. For example, aging countries should expect high mortality rates and implement additional active measures because the mortality is higher in elders (**Figure 2C**). Additionally, the current evidence illustrated that dogs and cats are susceptible to SARS-CoV-2 (**Figure 1E**) (Shi et al., 2020), and it is recommended that these pets be tested and isolated once their owners are infected. Despite hope for a vaccine, the possibility of antibody-dependent enhancement cannot be eliminated (Tetro, 2020). More, as the virus mutates, the protection of the vaccine diminishes (Zhou et al., 2021; Wang, P. et al., 2021). Vaccinees can also contract new virus strains, such as the latest Delta and Omicron outbreaks, according to open news. Although some medications may be effective, there are no cures for COVID-19 (Li et al., 2020). As a result, rather than passively waiting for vaccines or treatments, it is vital to take efficient containment measures. Because of the disease’s high rate of asymptomatic spread, the efforts of a single country or region will be insufficient to combat it (**Figure 3E**). Covert coronavirus infections could be seeding new outbreaks, which could result in periodic epidemics (Qiu, 2020). To prevent its rapid spread and let people return to their normal lives as soon as possible, collaborative efforts, including stronger environmental regulations, are required (Zhang et al., 2020). A more restrictive setting is especially helpful for preventing asymptomatic spread. According to an open report, an asymptomatic case in China’s Jilin province recently spread to over 100 people (People’s Daily, January 17, 2021).

Achieving zero-COVID is not easy, but the alternative is far worse (McKee, 2020). It’s worth noting that China aggressively implemented the zero-COVID plan precisely because it understood the serious threat of asymptomatic spread. China has reaped significant benefits from this method, making it the first large country in the world to fully recover from the epidemic. It achieves not only normal production and life but also positive economic growth. On the contrary, the virus continues to spread in some nations due to inadequate preventative efforts, increasing the risk of virus mutation and the development of harmful mutations, particularly the latest Delta and Omicron virus strains, making the outbreak deadly and uncontrollable. Admittedly, the feasibility of implementing these measures depends on the legal and cultural differences of different countries. We propose that science popularization be bolstered to enhance public knowledge of epidemic prevention. Countries having difficulty implementing these policies may pass temporary laws to alleviate the social crisis. Instead of large-scale lock-downs, a flexible, zero-COVID strategy would achieve success at a small cost and reduce its adverse impact on the economy. Due to the asymptomatic concealment, we must invest more in building more BSL-4 (Biosafety Level 4) laboratories with higher administrative standards, and invest more in research and technology to ascertain the reasons underlying asymptomatic infection and viral transmission. In reality, not every country has BSL-4 laboratories and the necessary funding and personnel to research highly lethal and contagious viruses. As a result, it is necessary to overcome political preconceptions and improve international cooperation and data sharing. It also necessitates bolstering healthcare infrastructure and more sensitive social sectors. Here, the revelation of the critical role of the asymptomatic transmission is a warning that a multipronged strategy is needed to avoid the syndemic health and the social and economic crisis we are suffering.

## Conclusion

Using extensive, systematic, and comparative assessments of SARS, MERS, and COVID-19, we discovered that COVID-19 has a lower mortality rate than SARS and MERS but a greater death toll and a higher proportion of asymptomatic cases. Asymptomatic transmissibility was also identified as one of the distinctive traits that led to the COVID-19 pandemic. Furthermore, we created a new operational software to mimic COVID-19 dissemination in Wuhan, which revealed a hundredfold increase in cumulative cases of existing asymptomatic spread compared to merely symptomatic spread. This reaffirmed the critical significance of asymptomatic transmissibility in COVID-19’s unchecked worldwide spread. In short, we here highlighted the triggering role of asymptomatic transmissibility in this unprecedented global crisis, which offers critical insights into the zero-COVID strategy against the recurring of COVID-19.

## Data Availability Statement

The original contributions presented in the study are included in the article/**Supplementary Material**. Further inquiries can be directed to the corresponding author.

## Author Contributions

CZ, XL designed the study and wrote the manuscript. CZ, HW, ZW, MG, LL performed data collection and analyzation. All authors contributed to the article and approved the submitted version.

## Funding

This work was supported by grants from the Outstanding Leaders Training Program of Pudong Health Bureau of Shanghai (PWRl2018-02), Pudong New Area Science and Technology Commission (PKJ2019-Y21), China Postdoctoral Science Foundation (2019M651615), Shanghai Super-Postdoctoral Science Foundation (2018229), National Natural Science Foundation of China (82101631).

## Conflict of Interest

The authors declare that the research was conducted in the absence of any commercial or financial relationships that could be construed as a potential conflict of interest.

## Publisher’s Note

All claims expressed in this article are solely those of the authors and do not necessarily represent those of their affiliated organizations, or those of the publisher, the editors and the reviewers. Any product that may be evaluated in this article, or claim that may be made by its manufacturer, is not guaranteed or endorsed by the publisher.
