# Supplementary material for: Asymptomatic Transmissibility Calls for Implementing a Zero-COVID Strategy to End the Current Global Crisis
Source: Front Cell Infect Microbiol. 2022 Apr 19;12:836409. doi: 10.3389/fcimb.2022.836409 (PMC9062041; doi:10.3389/fcimb.2022.836409)
Supplement: Supplementary file 1 [file DataSheet_1.pdf]

Supplementary S1-original data and references

Virus	Types of population	Location of study	Number of subjects	Description of subjects	Laboratory results	Number of asymptomatic subjects	Detection method	Author	Proportion of asymptomatic subjects	95% Credible interval（%）
SARS-CoV	General population	Hong Kong of China	828	Healthy blood donors，Non-pneumonic adult patients，Non-pneumonic paediatric patients，Symptom-free health-care workers		4	ELISA	Woo, P.C[1]		
		Hong Kong of China	938	Healthy adults		17	ELISA	Bo Jian Zheng[2]		
		Hong Kong of China	12000	General population in a community stricken by SARS		47	ELISA	D. T. M. Leung[3]		
		Sum	13766			68			0.49%（68/13766）	[0.39, 0.63]
	Contacts of confirmed cases	Guang Zhou of China	115	Contacts of confirmed cases		1	ELISA	Che, X. Y.[4]		
		Hong Kong of China	1068			2	ELISA	GM Leung[5]		
		France	37			0	ELISA	Le Vu, S[6]		
		Sum	1220			3			0.25%（3/1220）	[0.06, 0.78]
	HCWs	Hong Kong of China	131	HCWs		3	ELISA	Ip, M.[7]		
		Hong Kong of China	101	HCWs		1	ELISA	Lee, H. K.[8]		
		Taiwan of China	193	HCWs		1	ELISA	Wei-Tien Chang[9]		
		Hong Kong of China	647	HCWs		0	ELISA	Chan, P. K. [10]		
		Hong Kong of China	668	HCWs		1	ELISA	Lai, T. S. [11]		
		Taiwan of China	433	HCWs		4	ELISA	Hsueh, P. R.[12]		
		Singapore	80	HCWs		6	ELISA	Wilder-Smith, A.[13]		
		Bei jin of China	1127	HCWs		29	ELISA	Wang, Z. H.[14]		
		Guang Zhou of China	137	HCWs		4	ELISA	CDC; D Yu, MD[15]		
		Sum	3544			49	ELISA		1.38% (49/3544)	[1.04,1.84]

Virus	Types of population	Location of study	Number of subjects	Description of subjects	Laboratory results	Number of asymptomatic subjects	Detection method	Author	Proportion of asymptomatic subjects	
MERS-CoV	Occupational exposure to the host	Qatar	294	Occupational contact with dromedary camels	10	10	ELISA	Reusken, C. B.[16]		
		Kenya	1222	Livestock herders	2	2	ELISA	Liljander, A.[17]		
		Sum	1516			12			0.79% (12/1516)	[0.43, 1.42]
	General population	Western part of Saudi Ar	7461	Healthy adult blood donors		17	ELISA	Degnah A.A.[18]		
		Saudi Arabia	10009	Healthy individuals		15	ELISA	Muller, M. A.[19]		
		Qatar	4719	Healthy blood donors		1	ELISA	Al Kahlout, R. A.[20]		
		Sum	21649			33			0.15%(33/21649)	[0.11, 0.22]
	Contacts of confirmed cases	United Kingdom	33	Contacts of a confirmed case	2	0	rRT-PCR	Health Protection Agency[21]		
		France	162	Contacts of a confirmed case	1	0	rRT-PCR	Mailles, A.[22]		
		KSA	79	Contacts of a confirmed case	11	2	rRT-PCR	Arwady, M. A.[23]		
		United Araby Emirates	124	Contacts of a confirmed case	13	3	ELISA	Al Hosani, F. I.[24]		
		Qatar	135	Contacts of a confirmed case		1	ELISA	Al Kahlout, R. A.[20]		
		Korea	1610	Contacts of a confirmed case		7	ELISA	Yeong-Jun Song[25]		
		Sum	2143			13			0.61% (13/2143)	[0.34, 1.06]
	Confirmed suspected cases	United Arab Emirates	1586	Suspected MERS case patients	65	23	rRT-PCR	Al Hosani, F. I.[26]		
		KSA	57363	Suspected MERS case patients	384	19	rRT-PCR	Saeed, A. A.[27]		
		Sum			449	42			9.35% (42/449)	[6.90, 12.53]
	Laboratory confirmed cases	KSA		Confirmed MERS-CoV infection	255	64	rRT-PCR	Oboho, I. K.[28]		
		KSA		Confirmed MERS-CoV infection	38	2	rRT-PCR,ELISA	Assiri, A.[29]		
		KSA		Patient-contacts in hospital	61	3	rRT-PCR	Alenazi, T. H.[30]		
		KSA		MERS survivors	18	3	rRT-PCR,ELISA	Zhao, J.[31]		
		Jordan		Patient-contacts in hospital	16	3	rRT-PCR,ELISA	Payne, D. C.[32]		
				Confirmed MERS-CoV infection	144	18		WHO[33]		

		Sum			532	93			17.48% (93/532)	[14.40, 21.04]
	Confirmed HCWs	KSA		HCWs	18	6	rRT-PCR,ELISA,IFA	Alshukairi, A. N.[34]		
		KSA		HCWs	7	4	rRT-PCR,ELISA,IFA	Assiri, A.[29]		
		KSA		HCWs	43	25	rRT-PCR	Balkhy, H. H.[35]		
		United Arab Emirates		HCWs	31	12	rRT-PCR	Al Hosani, F. I.[26]		
		KSA		HCWs	43	18	rRT-PCR	Alenazi, T. H.[30]		
		Riyadh		HCWs	15	8	rRT-PCR	Amer, H.[36]		
		Global		HCWs	389	94		Grant, R.[37]		
		Sum			546	167			30.5% (167/546 ）	[26.78 , 34.67]
	WHO-Laboratory confirmed cases		2274	Laboratory confirmed MERS-CoV infection		298		Grant, R.[37]		
			642	Laboratory confirmed MERS-CoV infection		35		Elkholy, A. A.[38]		
		Sum	2916			333			11.42% (333/2916)	[10.30, 12.64]
	WHO-Confirmed HCWs				338	89		Elkholy, A. A.[38]		
			2223	HCWs	414	164		Grant, R.[37]		
		Sum			752	253			33.64% (253/752)	[30.29, 37.16]
COVID-19	Types of population	Location of study	Number of subjects	Description of subjects	Laboratory results	Number of asymptomatic subjects	Detection method	Author	Proportion of asymptomatic subjects	
	Other countries/areas	Japan	565	Evacuated population from China	9	5	rRT-PCR	Update to 2020-02-01[39]	55.56% (5/565）	
		Korea	701	Evacuated population from China	2	0	rRT-PCR	Update to 2020-02-04[40]	0	
		Germany	114	Evacuated population from China	2	1	rRT-PCR	Update to 2020-02-02[41]	0	
		Singapore	92	Evacuated population from China	4	2	rRT-PCR	Update to 2020-02-10[42]	50.00%(2/4)	
		France	434	Evacuated population from China	0	0	rRT-PCR	Update to 2020-02-08[43]	0	
		Iceland	2283	Random-Sample Population Screening	13	7	rRT-PCR	Update to 2020-04-04[44]		
		Sum	4189		30	15			0.36%(15/4189)	[0.21, 0.60]
	Other countries/areas	Japan	565	Evacuated population from China	9	5	rRT-PCR	Update to 2020-02-01[39]	55.56% (5/9）	
		South Korea	701	Evacuated population from China	2	0	rRT-PCR	Update to 2020-02-04[40]	0	
		Germany	114	Evacuated population from China	2	1	rRT-PCR	Update to 2020-02-02[41]	0	
		Singapore	92	Evacuated population from China	4	2	rRT-PCR	Update to 2020-02-10[42]	50.00%(2/4)	
		France	434	Evacuated population from China	0	0	rRT-PCR	Update to 2020-02-08[43]	0	
		Sum	1916		17	8			47.06%(8/17)	[23.86, 71.47]
	Diamond Princess	Diamond Princess cruise	4061	Confirmed cases	705	392	rRT-PCR	Update to 2020-02-15[45]	55.60%%(392/705 ）	
		Sum	4061		705	392			55.60%(392/705）	[51.84, 59.30]
	Contacts of confirmed cases	Ningbo of China	2147	Contacts of confirmed cases	110	22	rRT-PCR	Update to 2020-03-06[46]		
		Jinan of China	1455	Contacts of confirmed cases	28	3	rRT-PCR	Update to 2020-03-01[47]		
		Hubei partial areas of Ch	738	Contacts of confirmed cases	70	28	rRT-PCR	Update to 2020-02-23[48]		
		Total	4340		208	53	rRT-PCR		1.22% (61/4304)	[1.09, 1.83]
	Partial areas of China(Screening)	Ningbo of China	2147	Contacts of confirmed cases	110	22	rRT-PCR	Update to 2020-03-06[46]		
		Jinan of China	1455	Contacts of confirmed cases	28	3	rRT-PCR	Update to 2020-03-01[47]		
		Hubei partial areas of Ch	738	Contacts of confirmed cases	70	28	rRT-PCR	Update to 2020-02-23[48]		
		Total	4340		208	53	rRT-PCR		21.11% (61/289)	[16.64, 26.36]
	Confirmed cases	Iceland	22279	Targeted Testing	1321	126		Update to 2020-04-04[44]	9.54(126/1321)	[8.03, 11.28]
	Early stage of China	He Nan of China		Confirmed cases	168	2	rRT-PCR	Update to 2020-01-24[49]	1.19% (2/168）	[0.21,4.68]
		Zhe Jiang of China		Confirmed cases	85	1	rRT-PCR	Update to 2020-01-29[50]	1.18% (1/85)	[0.06, 7.23]
		Shan Xi of China		Confirmed cases	87	1	rRT-PCR	Update to 2020-01-30[51]	1.15% (1/87）	[0.06, 7.13]
		Total			340	4			1.18%(4/340)	[0.38, 3.19]
	Shan Dong of China(Partial screening)	Shan Dong of China		Confirmed cases	246	14	rRT-PCR	Update to 2020-02-02[52]	5.69% (14/246)	[3.27, 9.57]

Confirmed cases	Ning bo of China	2147	Confirmed cases	191	30	rRT-PCR	Update to 2020-02-02[46]	15.71%(30/191)	[11.01, 21.83]
Confirmed cases	Ning bo of China	2147	Contacts of confirmed cases	110	22	rRT-PCR	Update to 2020-02-02[46]	1.02%(22/2147)	[0.66, 0.157]
Confirmed casesContacts with asymptomatic infected cases	Ning bo of China	146	Confirmed cases	6		rRT-PCR	Update to 2020-02-02[46]		
Middle stage of China(cumulative data)	China	72341	Patient records	44627	889	rRT-PCR	Update to 2020-02-12[53]	1.99% (889/44627）	[1.87, 2.13]
Late stage of China(cumulative data)	China	82295		82295	6176		Update to 2020-04-14[54]	7.50%(6176/82295)	[7.32, 7.69]
Imported cases		1500	Imported cases	1500	337	rRT-PCR	Update to 2020-04-14[55]	22.47%(337/1500)	[20.39, 24.68]

Health care workers (HCWs); Enzyme-linked immunosorbent assay (ELISA); Real time Reverse Transcription-Polymerase Chain Reaction (rRT-PCR); Immunofluorescence assay (IFA);Kingdom of Saudi Arabia (KSA); WHO, world health organization

1. Woo PC, Lau SK, Tsoi HW, et al. Relative rates of non-pneumonic SARS coronavirus infection and SARS coronavirus pneumonia. Lancet 2004; 363(9412): 841-5.
2. Zheng BJ, Wong KH, Zhou J, et al. SARS-related virus predating SARS outbreak, Hong Kong. Emerg Infect Dis 2004; 10(2): 176-8.
3. Leung DT, van Maren WW, Chan FK, et al. Extremely low exposure of a community to severe acute respiratory syndrome coronavirus: false seropositivity due to use of bacterially derived antigens. J Virol 2006; 80(18): 8920-8.
4. Che XY, Di B, Zhao GP, et al. A patient with asymptomatic severe acute respiratory syndrome (SARS) and antigenemia from the 2003-2004 community outbreak of SARS in Guangzhou, China. Clin Infect Dis 2006; 43(1): e1-5.
5. Leung GM, Ho LM, Lam TH, Hedley AJ, Peiris JS. Prevalence of SARS-CoV antibody in all Hong Kong patient contacts. Hong Kong Med J 2009; 15 Suppl 9: 27-9.
6. Le Vu S, Yazdanpanah Y, Bitar D, Emmanuelli J, Bonmarin I, Desenclos JC. Absence of infection in asymptomatic contacts of index SARS case in France. Euro Surveill 2006; 11(1): 9-10.
7. Ip M, Chan PK, Lee N, et al. Seroprevalence of antibody to severe acute respiratory syndrome (SARS)-associated coronavirus among health care workers in SARS and non-SARS medical wards. Clin Infect Dis 2004; 38(12): e116-8.
8. Lee HK, Tso EY, Chau TN, Tsang OT, Choi KW, Lai TS. Asymptomatic severe acute respiratory syndrome-associated coronavirus infection. Emerg Infect Dis 2003; 9(11): 1491-2.
9. Chang WT, Kao CL, Chung MY, et al. SARS exposure and emergency department workers. Emerg Infect Dis 2004; 10(6): 1117-9.
10. Chan PK, Ip M, Ng KC, et al. Severe acute respiratory syndrome-associated coronavirus infection. Emerg Infect Dis 2003; 9(11): 1453-4.
11. Lai TS, Keung Ng T, Seto WH, Yam L, Law KI, Chan J. Low prevalence of subclinical severe acute respiratory syndrome-associated coronavirus infection among hospital healthcare workers in Hong Kong. Scand J Infect Dis 2005; 37(6-7): 500-3.
12. Hsueh PR, Kao CL, Lee CN, et al. SARS antibody test for serosurveillance. Emerg Infect Dis 2004; 10(9): 1558-62.
13. Wilder-Smith A, Teleman MD, Heng BH, Earnest A, Ling AE, Leo YS. Asymptomatic SARS coronavirus infection among healthcare workers, Singapore. Emerg Infect Dis 2005; 11(7): 1142-5.
14. Wang ZH, Nong Y, Lin JT, et al. [Covert infection of severe acute respiratory syndrome in health-care professionals and its relation to the workload and the type of work]. Zhonghua Jie He He Hu Xi Za Zhi 2004; 27(3): 151-4.
15. D Yu M, H Li, R Xu, MPH, J He, J Lin, L Li, W Li, H Xu, S Huang, J Huang. Prevalence of IgG Antibody to SARS-Associated Coronavirus in Animal Traders --- Guangdong Province, China, 2003. Guangdong Center for Disease Control 2003: https://www.cdc.gov/mmwr/preview/mmwrhtml/mm5241a2.htm.
16. Reusken CB, Farag EA, Haagmans BL, et al. Occupational Exposure to Dromedaries and Risk for MERS-CoV Infection, Qatar, 2013-2014. Emerg Infect Dis 2015; 21(8): 1422-5.
17. Liljander A, Meyer B, Jores J, et al. MERS-CoV Antibodies in Humans, Africa, 2013-2014. Emerg Infect Dis 2016; 22(6): 1086-9.
18. Degnah AA, Al-Amri SS, Hassan AM, et al. Seroprevalence of MERS-CoV in healthy adults in western Saudi Arabia, 2011-2016. J Infect Public Health 2020.
19. Muller MA, Meyer B, Corman VM, et al. Presence of Middle East respiratory syndrome coronavirus antibodies in Saudi Arabia: a nationwide, cross-sectional, serological study. Lancet Infect Dis 2015; 15(6): 629.
20. Al Kahlout RA, Nasrallah GK, Farag EA, et al. Comparative Serological Study for the Prevalence of Anti-MERS Coronavirus Antibodies in High- and Low-Risk Groups in Qatar. J Immunol Res 2019; 2019: 1386740.
21. Health Protection Agency UKNCIt. Evidence of person-to-person transmission within a family cluster of novel coronavirus infections, United Kingdom, February 2013. Euro Surveill 2013; 18(11): 20427.
22. Mailles A, Blanckaert K, Chaud P, et al. First cases of Middle East Respiratory Syndrome Coronavirus (MERS-CoV) infections in France, investigations and implications for the prevention of human-to-human transmission, France, May 2013. Euro Surveill 2013; 18(24).
23. Arwady MA, Alraddadi B, Basler C, et al. Middle East Respiratory Syndrome Coronavirus Transmission in Extended Family, Saudi Arabia, 2014. Emerg Infect Dis 2016; 22(8): 1395-402.
24. Al Hosani FI, Kim L, Khudhair A, et al. Serologic Follow-up of Middle East Respiratory Syndrome Coronavirus Cases and Contacts-Abu Dhabi, United Arab Emirates. Clin Infect Dis 2019; 68(3): 409-18.
25. Song YJ, Yang JS, Yoon HJ, et al. Asymptomatic Middle East Respiratory Syndrome coronavirus infection using a serologic survey in Korea. Epidemiol Health 2018; 40: e2018014.
26. Al Hosani FI, Pringle K, Al Mulla M, et al. Response to Emergence of Middle East Respiratory Syndrome Coronavirus, Abu Dhabi, United Arab Emirates, 2013-2014. Emerg Infect Dis 2016; 22(7): 1162-8.
27. Saeed AA, Abedi GR, Alzahrani AG, et al. Surveillance and Testing for Middle East Respiratory Syndrome Coronavirus, Saudi Arabia, April 2015-February 2016. Emerg Infect Dis 2017; 23(4): 682-5.
28. Oboho IK, Tomczyk SM, Al-Asmari AM, et al. 2014 MERS-CoV outbreak in Jeddah--a link to health care facilities. N Engl J Med 2015; 372(9): 846-54.
29. Assiri A, Abedi GR, Bin Saeed AA, et al. Multifacility Outbreak of Middle East Respiratory Syndrome in Taif, Saudi Arabia. Emerg Infect Dis 2016; 22(1): 32-40.
30. Alenazi TH, Al Arbash H, El-Saed A, et al. Identified Transmission Dynamics of Middle East Respiratory Syndrome Coronavirus Infection During an Outbreak: Implications of an Overcrowded Emergency Department. Clin Infect Dis 2017; 65(4): 675-9.
31. Zhao J, Alshukairi AN, Baharoon SA, et al. Recovery from the Middle East respiratory syndrome is associated with antibody and T-cell responses. Sci Immunol 2017; 2(14).
32. Payne DC, Biggs HM, Al-Abdallat MM, et al. Multihospital Outbreak of a Middle East Respiratory Syndrome Coronavirus Deletion Variant, Jordan: A Molecular, Serologic, and Epidemiologic Investigation. Open Forum Infect Dis 2018; 5(5): ofy095.
33. Who Mers-Cov Research G. State of Knowledge and Data Gaps of Middle East Respiratory Syndrome Coronavirus (MERS-CoV) in Humans. PLoS Curr 2013; 5.
34. Alshukairi AN, Khalid I, Ahmed WA, et al. Antibody Response and Disease Severity in Healthcare Worker MERS Survivors. Emerg Infect Dis 2016; 22(6).
35. Balkhy HH, Alenazi TH, Alshamrani MM, et al. Description of a Hospital Outbreak of Middle East Respiratory Syndrome in a Large Tertiary Care Hospital in Saudi Arabia. Infect Control Hosp Epidemiol 2016; 37(10): 1147-55.
36. Amer H, Alqahtani AS, Alaklobi F, Altayeb J, Memish ZA. Healthcare worker exposure to Middle East respiratory syndrome coronavirus (MERS-CoV): Revision of screening strategies urgently needed. Int J Infect Dis 2018; 71: 113-6.
37. Grant R, Malik MR, Elkholy A, Van Kerkhove MD. A review of asymptomatic and sub-clinical Middle East Respiratory Syndrome Coronavirus Infections. Epidemiol Rev 2019.
38. Elkholy AA, Grant R, Assiri A, Elhakim M, Malik MR, Van Kerkhove MD. MERS-CoV infection among healthcare workers and risk factors for death: Retrospective analysis of all laboratory-confirmed cases reported to WHO from 2012 to 2 June 2018. J Infect Public Health 2019.
39. Nishiura H, Kobayashi T, Yang Y, et al. The Rate of Underascertainment of Novel Coronavirus (2019-nCoV) Infection: Estimation Using Japanese Passengers Data on Evacuation Flights. J Clin Med 2020; 9(2).

39. Nishiura H, Kobayashi T, Yang Y, et al. The Rate of Underascertainment of Novel Coronavirus (2019-nCoV) Infection: Estimation Using Japanese Passengers Data on Evacuation Flights. J Clin Med 2020; 9(2).
40. Cooperation DoRaaI. Enhanced the 2019-nCoV quarantine guideline in Republic of Korea. 2020-02-03 2020: https://www.cdc.go.kr/board/board.es?mid=a30402000000&bid=0030&tag=&act=view&list_no=365953.
41. Hoehl S, Berger A, Kortenbusch M, et al. Evidence of SARS-CoV-2 Infection in Returning Travelers from Wuhan, China. N Engl J Med 2020.
42. network TST-An. Coronavirus: All 92 evacuated from Wuhan on Scoot flight being tested. https://asianewsnetwork/2020/02/10/coronavirus-in-singapore-of-the-43-cases-so-far-26-are-male-17-female-local-cases-now-exceed-imported-ones/ 2020-02-10.
43. news Y. Coronavirus tests on two French citizens evacuated from China turn out negative. Coronavirus: French evacuees from China test negative for virus. https://uknewsyahoocom/plane-carrying-french-citizens-virus-123813390html?guccounter=1&guce_referrer=aHR0cHM6Ly9jbi5iaW5nLmNvbS8&guce_referrer_sig=AQAAAKYYTbyq2KdaFzo_-3ILtoPYPI_es_PwDz3dUP4PbJArlqQ2W5pQ22bQTij_NAEHlU887ftQUspq1-x6CzGN3t0yUywPRvDcRP6LfF6iDkp3wI_MDCGdyDSB3YXbYcb2WKO4bhMRkPxr60_ZdnfPfdLzLnqQ0m_dJs6eWbJ5x-ta 2020-01-31.
44. Gudbjartsson DF, Helgason A, Jonsson H, et al. Spread of SARS-CoV-2 in the Icelandic Population. N Engl J Med 2020.
45. News S. COVID Confirmed Cases On Diamond Princess Increases to 705. https://recombinomicsco/topic/4973-covid-confirmed-cases-on-diamond-princess-increases-to-705/ 2020-01-16.
46. Chen Yi WA, Yi Bo, Ding Keqin, Wang Haibo, Wang Jianmei, Shi Hongbo, Wang Sijia, Xu, Guozhang. The epidemiological characteristics of infection in close contacts of COVID-19 in Ningbo city. Chinese Journal of Epidemiology 2020; 41(41): 1-7.
