# Supplementary material for: Asymptomatic Transmissibility Calls for Implementing a Zero-COVID Strategy to End the Current Global Crisis
Source: Front Cell Infect Microbiol. 2022 Apr 19;12:836409. doi: 10.3389/fcimb.2022.836409 (PMC9062041; doi:10.3389/fcimb.2022.836409)
Supplement: Supplementary file 2 [file DataSheet_2.pdf]

| Supplementary S2 includes 6 tables | |
|---|---|
| Table-S0 | Supplementary S1 information |
| Table-S1-Fig.1 | Fig1.A-E: raw data and references |
| Table-S1-Fig.2 | Fig1.A-E: raw data and references |
| Table-S1-Fig.3 | Fig1.A-E: raw data and references |
| Table-S1-Fig.4 | Fig1.A-E: raw data and references |
| Table-S1-Fig.5 | Fig1.A-E: raw data and references |

**Table-S1-Fig.1**

Fig.1.C-D

| | SRAS-CoV(Update 20030807) | MERS-CoV(Update 20190901) | COVID-19(Update 20200416) | | References | | |
|---|---|---|---|---|---|---|---|
| Infection cases | 8422 | 2494 | 1991562 | | 1,2,3,4 | | |
| Epidemic time (Month) | 12 | 74 | 4 | | 1,2,3,4 | | |
| Countries or areas reported cases globally | 30 | 27 | 213 | | 1,2,3,4 | | |
| Spread speed in areas or countries | 2.5(30/12) | 0.36(27/74) | 53.25(213/4) | | 1,2,3,4 | | |
| Death | 916 | 858 | 130885 | | 1,2,3,4 | | |
| Death speed per month | 76.33(8422/12) | 11.59(858/74) | 32721.25(1991562/4) | | 1,2,3,4 | | |
| Fatality ratio | 10.88%(916/8422) | 34.40%(858/2494) | 6.57%(130885/1991562) | | 1,2,3,4 | | |

| | | | | | | | |
|---|---|---|---|---|---|---|---|
| COVID-19 infected cases (Update 20200416) | COVID-19 Death | COVID-19 Confirmed cases | Fatality ratio in infected cases(%) | Population （Ten thousand) | COVID-19 Infection ratio（Per 10000 | | References |
| China | 4642 | 84149 | 5.52 | 140000 | 0.60 | update to 202004117 | 3 |
| United States of America | 28221 | 632781 | 4.46 | 33000 | 19.18 | update to 202004117 | 3 |
| Japan | 148 | 9169 | 1.61 | 12477.6 | 0.73 | update to 202004117 | 3 |
| Germany | 3867 | 133830 | 2.89 | 8292.7 | 16.14 | update to 202004117 | 3 |
| Iran | 4869 | 77995 | 6.24 | 8165 | 9.55 | update to 202004117 | 3 |
| France | 17899 | 107778 | 16.61 | 6698.7 | 16.09 | update to 202004117 | 3 |
| The United Kingdom | 13729 | 103097 | 13.32 | 6648.9 | 15.51 | update to 202004117 | 3 |
| Italy | 22172 | 168941 | 13.12 | 6043 | 27.96 | update to 202004117 | 3 |
| South Korea | 230 | 10635 | 2.16 | 5163.5 | 2.06 | | 3 |

| | | | | | | | |
|---|---|---|---|---|---|---|---|
| Spain | 19130 | 182816 | 10.46 | 4673 | 39.12 | update to 202004117 | 3 |
| Singapore | 10 | 4427 | 0.23 | 564 | 7.85 | update to 202004117 | 3 |
| Iceland | 8 | 1739 | 0.46 | 35.3 | 49.26 | update to 202004117 | 3 |
| Diamond Princess cruise ship | 10 | 721 | 1.39 | | | | |

Fig.1.A

| Name | References |
|---|---|
| HCoV-229E,HCoV-HKU1, HCoV-NL63, HCoV-OC43 | 5,6 |
| MERS-CoV, SARS-CoV,COVID-19 | 5,6,7 |

Fig.1.E

| Name | Infection species | References |
|---|---|---|
| SRAS-CoV | Human, civet, cat, fe | 8,9 |
| MERS-CoV | Human bat, dromeda | 10,11 |
| COVID-19 | Human, cat, ferret , ( | 12,13 |


1. WHO. Summary of probable SARS cases with onset of illness from 1 November 2002 to 31 July 2003. 20031231 2003: https://www.who.int/csr/sars/country/table2004_04_21/en/.
2. WHO. MERS situation update, January 2020. 20200101 2020: http://www.emro.who.int/pandemic-epidemic-diseases/mers-cov/mers-situation-update-january-2020.html.
3. WHO. Coronavirus disease 2019 (COVID-19) Situation Report – 88. 20200416 2020: https://www.who.int/docs/default-source/coronaviruse/situation-reports/20200417-sitrep-88-covid-191b6cccd94f8b4f219377bff55719a6ed.pdf?sfvrsn=ebe78315_6.
4. WHO. Chapter 5: SARS: lessons from a new disease. 20031231 2003: https://www.who.int/whr/2003/chapter5/en/index2.html.
5. Su S, Wong G, Shi W, et al. Epidemiology, Genetic Recombination, and Pathogenesis of Coronaviruses. Trends Microbiol 2016; 24(6): 490-502.
6. Gaunt ER, Hardie A, Claas EC, Simmonds P, Templeton KE. Epidemiology and clinical presentations of the four human coronaviruses 229E, HKU1, NL63, and OC43 detected over 3 years using a novel multiplex

real-time PCR method. J Clin Microbiol 2010; 48(8): 2940-7.
7. WHO. Coronavirus disease (COVID-19) Pandemic. 2020:
https://www.who.int/emergencies/diseases/novel-coronavirus-2019.
8. Drosten C, Gunther S, Preiser W, et al. Identification of a novel coronavirus in patients with severe acute respiratory syndrome. N Engl J Med 2003; 348(20): 1967-76.
9. Martina BE, Haagmans BL, Kuiken T, et al. Virology: SARS virus infection of cats and ferrets. Nature 2003; 425(6961): 915.
10. Bermingham A, Chand MA, Brown CS, et al. Severe respiratory illness caused by a novel coronavirus, in a patient transferred to the United Kingdom from the Middle East, September 2012. Euro Surveill 2012; 17(40): 20290.
11. Reusken CB, Schilp C, Raj VS, et al. MERS-CoV Infection of Alpaca in a Region Where MERS-CoV is Endemic. Emerg Infect Dis 2016; 22(6): 1129-31.
12. Lam TT, Shum MH, Zhu HC, et al. Identifying SARS-CoV-2 related coronaviruses in Malayan pangolins. Nature 2020.
13. Shi J, Wen Z, Zhong G, et al. Susceptibility of ferrets, cats, dogs, and other domesticated animals to SARS-coronavirus 2. Science 2020.

## Table-S1-Fig.2

| Fig.2.A | | | | | |
|---|---|---|---|---|---|
| SARS-CoV | Asymptc | Mild | Severe | | References |
| Beijing | 0 | 169 | 81 | | 2 |
| Hongkong | 0 | 106 | 32 | | 3 |
| Singapore | | 153 | 46 | | 4 |
| Toronto | | 116 | 38 | | |
| Total | 0 | 544 | 197 | 741 | |
| | | 73.41%(544/741 | 26.59%(197/74 | 1) | |

| | | | | | |
|---|---|---|---|---|---|
| MERS-CoV | Asymptc | Mild | Severe | | |
| | 82 | 148 | 67 | | 5 |
| | 8 | 7 | 0 | | |
| Total | 245 | | 67 | 312 | |
| | | 78.53%(245/312 | 21.47%(67/312 | ) | |
| | | | | | |
| COVID19 | Asymptc | Mild | Severe | | 1 |
| | 0 | 36160 | 8255 | | |
| Total | 36160 | | 8255 | 44415 | |
| | | 81.41%(36160/4 | 18.59%(8255/4 | 4415) | |

| Fig.2.B-C |
|---|
| References: Table-S1-Fig.1 data |

| Fig.2.D | | | | | | |
|---|---|---|---|---|---|---|
| SARS-CoV | Age | SARS-CoV infection cases | SARS-CoV death cases | Infection ratio(%) | Death ratio(%) | References |
| | <19 | 300 | 3 | 7.92 | 1.00 | Fig.2.A SARS-CoV detail data |
| | 20-29 | 1134 | 12 | 29.93 | 1.06 | |
| | 30-39 | 877 | 24 | 23.15 | 2.74 | |
| | 40-49 | 676 | 42 | 17.84 | 6.21 | |
| | 50-59 | 351 | 45 | 9.26 | 12.82 | |
| | 60-69 | 242 | 48 | 6.39 | 19.83 | |
| | >70 | 209 | 66 | 5.52 | 31.58 | |
| | Total | 3789 | 240 | | | |

| MERS-nCoV | Age | MERS-nCoV-infection cases in South Korea | MERS-nCoV-death cases in South Korea | Infection ratio(%) | Death ratio(%) | References |
|---|---|---|---|---|---|---|
| | <19 | 4 | 0 | 1.72 | 0.00 | Fig.2.A MERS-CoV detail data |
| | 20-29 | 19 | 0 | 8.15 | 0.00 | |
| | 30-39 | 38 | 2 | 16.31 | 5.26 | |
| | 40-49 | 32 | 1 | 13.73 | 3.13 | |
| | 50-59 | 52 | 12 | 22.32 | 23.08 | |
| | 60-69 | 42 | 15 | 18.03 | 35.71 | |
| | >70 | 46 | 24 | 19.74 | 52.17 | |
| | Total | 233 | 54 | | | |

| COVID-19 | Age | COVID-19-infection cases in China | COVID−19 death cases in China | Infection ratio(%) | Death ratio(%) | References |
|---|---|---|---|---|---|---|
| | <19 | 965 | 1 | 2.16 | 0.10 | 1 |
| | 20-29 | 3619 | 7 | 8.10 | 0.19 | |
| | 30-39 | 7600 | 18 | 17.01 | 0.24 | |
| | 40-49 | 8571 | 38 | 19.19 | 0.44 | |
| | 50-59 | 10008 | 130 | 22.40 | 1.30 | |
| | 60-69 | 8583 | 309 | 19.21 | 3.60 | |
| | >70 | 5326 | 520 | 11.92 | 9.76 | |
| | Total | 44672 | 1023 | | | |

| |
|---|
| Fig.2.E |
| References: Table-S1-Fig.1 data |

Fig.2.A MERS-CoV detail data

| MERS-nCoV | | Infection cases in Jordan | Death cases in Jordan | Infection cases in Saudi Arabia | Death cases in Saudi Arabia | Infection cases | Death cases | Infection cases in South Korea | Death cases in South Korea | Total Infection | Total Death | Death ratio(%) | References |
|---|---|---|---|---|---|---|---|---|---|---|---|---|---|
| | <19 | 2 | 0 | 1 | 0 | | 0 | 1 | 0 | 4 | 0 | 0 | 6 |

| | | | | | | | | | | | | | |
|---|---|---|---|---|---|---|---|---|---|---|---|---|---|
| | 20-29 | 1 | 0 | | 0 | 5 | 0 | 13 | 0 | 19 | 0 | 0 | 7 |
| | 30-39 | 2 | 0 | 1 | 1 | 9 | 0 | 26 | 1 | 38 | 2 | 5.26 | 8 |
| | 40-49 | 1 | 0 | 1 | 0 | 1 | 0 | 29 | 1 | 32 | 1 | 3.13 | |
| | 50-59 | 3 | 3 | 7 | 3 | | 0 | 42 | 6 | 52 | 12 | 23.08 | |
| | 60-69 | 4 | 2 | 2 | 2 | | 0 | 36 | 11 | 42 | 15 | 35.71 | |
| | >70 | 3 | 2 | 4 | 4 | | 0 | 39 | 18 | 46 | 24 | 52.17 | |
| | | 16 | 7 | 16 | 10 | 15 | 0 | 186 | 37 | 233 | 54 | | |

Fig.2.A SARS-CoV detail data

| SARS-nCoV | Age | SARS-nCoV Beijin infection cases inChina | SARS-nCoV Beijin death cases in China | | | SARS-nCoV Guangzhou infection cases in China | SARS-nCoV Guangzhou death cases inChina | Total infection cases | Infection ratio(%) | Total death cases | Death ratio(%) | References |
|---|---|---|---|---|---|---|---|---|---|---|---|---|
| | <19 | 189 | 1 | | <19 | 111 | 2 | 300 | 7.92 | 3 | 1.00 | 2,9 |
| | 20-29 | 781 | 10 | | 20-29 | 353 | 2 | 1134 | 29.93 | 12 | 1.06 | |
| | 30-39 | 567 | 19 | | 30-39 | 310 | 5 | 877 | 23.15 | 24 | 2.74 | |
| | 40-49 | 474 | 32 | | 40-49 | 202 | 10 | 676 | 17.84 | 42 | 6.21 | |
| | 50-59 | 228 | 38 | | 50-59 | 123 | 7 | 351 | 9.26 | 45 | 12.82 | |
| | 60-69 | 149 | 37 | | 60-69 | 93 | 11 | 242 | 6.39 | 48 | 19.83 | |
| | >70 | 130 | 55 | | >70 | 79 | 11 | 209 | 5.52 | 66 | 31.58 | |
| Total | | | | | | | | 3789 | | | | |


1. Novel Coronavirus Pneumonia Emergency Response Epidemiology T. [The epidemiological characteristics of an outbreak of 2019 novel coronavirus diseases (COVID-19) in China]. Zhonghua Liu Xing Bing Xue Za Zhi 2020; 41(2): 145-51.
2. Chen Qi LM, Liang Wangnian1. Analysis on fatality ratio of severe acute respiratory syndromes(SARS) in Beijing. Chinese Journal of Public Health 2004; 20(2): 134-5.
3. Lee N, Hui D, Wu A, et al. A major outbreak of severe acute respiratory syndrome in Hong Kong. N Engl J Med 2003; 348(20): 1986-94.
4. Lew TW, Kwek TK, Tai D, et al. Acute respiratory distress syndrome in critically ill patients with severe acute respiratory syndrome. JAMA 2003; 290(3): 374-80.
5. Al Hosani FI, Pringle K, Al Mulla M, et al. Response to Emergence of Middle East Respiratory Syndrome Coronavirus, Abu Dhabi, United Arab Emirates, 2013-2014. Emerg Infect Dis 2016; 22(7): 1162-8.
6. Butt TS, Koutlakis-Barron I, AlJumaah S, AlThawadi S, AlMofada S. Infection control and prevention practices implemented to reduce transmission risk of Middle East respiratory syndrome-coronavirus in a tertiary care institution in Saudi Arabia. Am J Infect Control 2016; 44(5): 605-11.
7. Payne DC, Biggs HM, Al-Abdallat MM, et al. Multihospital Outbreak of a Middle East Respiratory Syndrome Coronavirus Deletion Variant,

Jordan: A Molecular, Serologic, and Epidemiologic Investigation. Open Forum Infect Dis 2018; 5(5): ofy095.
8. Kim KH, Tandi TE, Choi JW, Moon JM, Kim MS. Middle East respiratory syndrome coronavirus (MERS-CoV) outbreak in South Korea, 2015: epidemiology, characteristics and public health implications. J Hosp Infect 2017; 95(2): 207-13.
9. Peng Guuowen HJ, Lin Jinyan. Epidemiological study of infectious atypical pneumonia in Guangdong Province Guangdong Medical Journal 2003; suppl(1): 36-8.

## Table-S1-Fig.3

| | SRAS-CoV | MERS-CoV | COVID-19 | References |
|---|---|---|---|---|
| Days for max virus loads after onset | 7-10[1,4,5] | 3-13[6,8] | -0.7-6[1,2,3, 7] | |
| Max virus loads in the upper respiratory tract(log10) | 5.5-8[4] | 5-6.6[6,8] | 6.5-8.85[1] | |
| Basic reproduction number(R0) | 2-5 | 2.5-8.09 | 1.5-6.9 | 8,9,10,11 |
| The average Incubation period (Day) | 4.3-5.1 | 4.5-7.8 | 4.4-5.5 | 12,13,14 ,14,15 |
| Maximum incubation period (Day) | 20[16] | 21[17] | 27[18] | |
| | | | | |
| Infection rate of close contacts of asymptomatic 2019-nCoV carriers (%) | 0 | 0 | 4.1[19] | |
| | | | | |

| | | | |
|---|---|---|---|
| Fig.3B | 20,21 | | |
| | | | |


1. Al-Tawfiq JA. Viral loads of SARS-CoV, MERS-CoV and SARS-CoV-2 in respiratory specimens: What have we learned? Travel Med Infect Dis 2020: 101629.
2. Peiris JS, Chu CM, Cheng VC, et al. Clinical progression and viral load in a community outbreak of coronavirus-associated SARS pneumonia: a prospective study. Lancet 2003; 361(9371): 1767-72.
3. Drosten C, Chiu LL, Panning M, et al. Evaluation of advanced reverse transcription-PCR assays and an alternative PCR target region for detection of severe acute respiratory syndrome-associated coronavirus. J Clin Microbiol 2004; 42(5): 2043-7.
4. Oh MD, Park WB, Choe PG, et al. Viral Load Kinetics of MERS Coronavirus Infection. N Engl J Med 2016; 375(13): 1303-5.
5. Corman VM, Albarrak AM, Omrani AS, et al. Viral Shedding and Antibody Response in 37 Patients With Middle East Respiratory Syndrome Coronavirus Infection. Clin Infect Dis 2016; 62(4): 477-83.
6. He X, Lau EHY, Wu P, et al. Temporal dynamics in viral shedding and transmissibility of COVID-19. Nat Med 2020.
7. Liu Y, Gayle AA, Wilder-Smith A, Rocklov J. The reproductive number of COVID-19 is higher compared to SARS coronavirus. J Travel Med 2020; 27(2).
8. Li Q, Guan X, Wu P, et al. Early Transmission Dynamics in Wuhan, China, of Novel Coronavirus-Infected Pneumonia. N Engl J Med 2020; 382(13): 1199-207.
9. Chang HJ. Estimation of basic reproduction number of the Middle East respiratory syndrome coronavirus (MERS-CoV) during the outbreak in South Korea, 2015. Biomed Eng Online 2017; 16(1): 79.
10. Wu JT, Leung K, Leung GM. Nowcasting and forecasting the potential domestic and international spread of the 2019-nCoV outbreak originating in Wuhan, China: a modelling study. Lancet 2020; 395(10225): 689-97.
11. Sanche S, Lin YT, Xu C, Romero-Severson E, Hengartner N, Ke R. High Contagiousness and Rapid Spread of Severe Acute Respiratory

[illegible]
Syndrome Coronavirus 2. Emerg Infect Dis 2020; 26(7).
12. Jiang X, Rayner S, Luo MH. Does SARS-CoV-2 has a longer incubation period than SARS and MERS? J Med Virol 2020; 92(5): 476-8.
13. Lessler J, Reich NG, Brookmeyer R, Perl TM, Nelson KE, Cummings DA. Incubation periods of acute respiratory viral infections: a systematic review. Lancet Infect Dis 2009; 9(5): 291-300.
14. Lauer SA, Grantz KH, Bi Q, et al. The Incubation Period of Coronavirus Disease 2019 (COVID-19) From Publicly Reported Confirmed Cases: Estimation and Application. Ann Intern Med 2020.
15. Park JE, Jung S, Kim A, Park JE. MERS transmission and risk factors: a systematic review. BMC Public Health 2018; 18(1): 574.
16. Lu H, Huo N, Xu X, et al. [The epidemiologic characteristics of patients with severe acute respiratory syndrome (SARS)]. Beijing Da Xue Xue Bao Yi Xue Ban 2003; 35 Suppl: 8-11.
17. Virlogeux V, Fang VJ, Park M, Wu JT, Cowling BJ. Comparison of incubation period distribution of human infections with MERS-CoV in South Korea and Saudi Arabia. Sci Rep 2016; 6: 35839.
18. Laguipo ABB. Coronavirus incubation period could be 27 days, longer than previously thought. 20200324 2020: https://www.news-medical.net/news/20200224/Coronavirus-incubation-period-could-be-27-days-longer-than-previously-thought.aspx.
19. Chen Yi WA, Yi Bo, Ding Keqin, Wang Haibo, Wang Jianmei, Shi Hongbo, Wang Sijia, Xu, Guozhang. The epidemiological characteristics of infection in close contacts of COVID-19 in Ningbo city. Chinese Journal of Epidemiology 2020; 41(41): 1-7.
20. Guo WL, Jiang Q, Ye F, et al. Effect of throat washings on detection of 2019 novel coronavirus. Clin Infect Dis 2020.
21. Wolfel R, Corman VM, Guggemos W, et al. Virological assessment of hospitalized patients with COVID-2019. Nature 2020.

## Table-S1-Fig.4

| | | | | | |
|---|---|---|---|---|---|
| Fig4.A-D | Supplementary S1 | | | | |

| Fig4.E | | | | | |
|---|---|---|---|---|---|
| Name | Infected cases | HCWs infection cases | HCWs infection ratio in infected cases(%) | | References |
| SRAS-CoV(Update 20030807) | 8096 | 1706 | 21.07 | | 4 |
| MERS-CoV(Update 20190901) | 2223 | 415 | 18.67 | | 5 |
| COVID-19(Update 20200327) | 189320 | 18668 | 9.86 | | Fig.4.E COVID-19 detail data |

| | | | | | |
|---|---|---|---|---|---|
| Fig4.F | Supplementary S1 | | | | |

| Fig.4.E COVID-19 detail data | | | | | |
|---|---|---|---|---|---|
| | Confirmed cases | HCWs infection cases | HCWs infection ratio in confirmed cases(%) | | |
| Spain | 64059 | 9444 | 14.74 | Update 20200327 | 1 |
| Italy | 80589 | 6205 | 7.70 | Update 20200327 | 2 |
| China | 44672 | 3019 | 6.76 | Update 20200223 | 3 |
| Total | 189320 | 18668 | 9.86 | | |
| | | | | | |

1. Spain HCWs infection ratio in 2020: https://www.theargus.co.uk/news/national/18341065.italy-passes-chinese-virus-infection-total-deaths-spain-surge/.
2. Italy HCWs infection ratio. 2020: https://www.dailymail.co.uk/news/article-8155987/37-Italian-doctors-died-coronavirus-6-205-medical-workers-infected.html.
3. China HCWs infection ratio. 2020: http://news.hexun.com/2020-02-20/200348624.html.
4. WHO. Summary of probable SARS cases with onset of illness from 1 November 2002 to 31 July 2003. 2003: https://www.who.int/csr/sars/country/table2004_04_21/en/.
5. Elkholy AA, Grant R, Assiri A, Elhakim M, Malik MR, Van Kerkhove MD. MERS-CoV infection among healthcare workers and risk factors for death: Retrospective analysis of all laboratory-confirmed cases reported to WHO from 2012 to 2 June 2018. J Infect Public Health 2020; 13(3): 418-22.

**Table-S1-Fig.5**

| Date | Native new confirmed cases in China | New imported confirmed cases in China | Native new asymptomatic cases in China | New imported asymptomatic cases in China | Confirmed cases transformed from asymptomatic infection | References |
|---|---|---|---|---|---|---|
| 4-01 | 0 | 35 | 38 | 17 | 9 | http://www.nhc.gov.cn/xcs/yqfkdt/gzbd_index.shtml |
| 4-02 | 2 | 29 | 53 | 7 | 7 | |
| 4-03 | 1 | 18 | 38 | 26 | 3 | |
| 4-04 | 5 | 25 | 31 | 16 | 3 | |
| 4-05 | 1 | 38 | 38 | 40 | 5 | |
| 4-06 | 0 | 32 | 21 | 9 | 2 | |
| 4-07 | 3 | 59 | 35 | 102 | 11 | |
| 4-08 | 2 | 61 | 28 | 28 | 15 | |
| 4-09 | 4 | 38 | 33 | 14 | 14 | |
| 4-10 | 4 | 42 | 27 | 7 | 14 | |
| 4-11 | 2 | 97 | 51 | 12 | 14 | |
| 4-12 | 10 | 98 | 49 | 12 | 28 | |
| 4-13 | 3 | 86 | 49 | 5 | 67 | |
| 4-14 | 10 | 36 | 54 | 3 | 7 | |
| 4-15 | 12 | 34 | 61 | 3 | 6 | |
| 4-16 | 11 | 15 | 63 | 3 | 10 | |
| 4-17 | 10 | 17 | 51 | 3 | 9 | |
| 4-18 | 9 | 7 | 41 | 3 | 0 | |
| 4-19 | 4 | 8 | 44 | 5 | 0 | |
| 4-20 | 7 | 4 | 35 | 2 | 3 | |
| Total | 100 | 779 | 840 | 317 | 227 | |
| Total infection cases | | 879 | | 1157 | | |
