# Supplementary material for: Asymptomatic Transmissibility Calls for Implementing a Zero-COVID Strategy to End the Current Global Crisis
Source: Front Cell Infect Microbiol. 2022 Apr 19;12:836409. doi: 10.3389/fcimb.2022.836409 (PMC9062041; doi:10.3389/fcimb.2022.836409)
Supplement: Supplementary file 3 [file DataSheet_3.pdf]

百度首页 moyuanmoy...

吉林1传102，无症状感染者为何能传染这么多人？

人民日报
发布时间: 2021-01-17 17:58 人民日报社 关注

2021年1月13日，吉林省卫健委通报1例无症状感染者，谁都未曾预想到这位黑龙江省输入的无症状感染者，会成为接下来4天疫情通报中的102例无症状感染者及确诊患者的感染关联源。

1月17日下午，吉林省疫情防控发布会通报，有102例感染者为同一输入病例林某传播。林某从事个体营销职业，黑龙江籍，近期多次往返于黑吉两省。

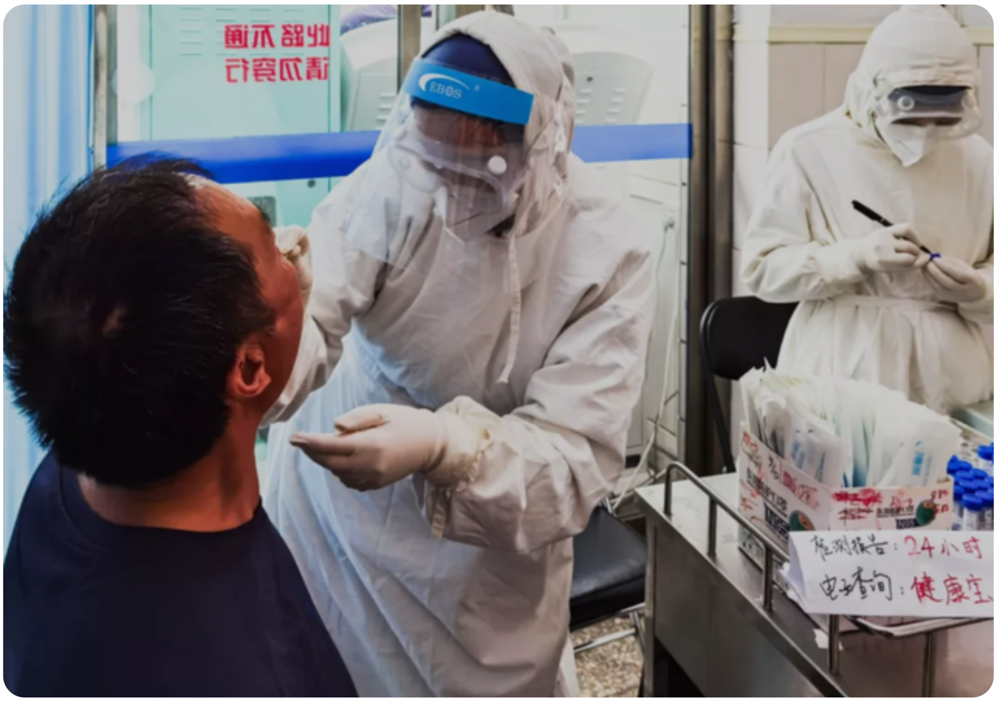

资料图

一条由无症状感染者引发的传播链条

1月5日，45岁的黑龙江籍个体营销人员林某，从黑龙江南岔乘K350次列车（11车13号）抵达哈尔滨西站。距离他座位不远的11车27号和28号座位，是一对从绥化市望奎县返回长春的中年夫妻。

1月11日，中年夫妻被确定为无症状感染者。1月12日，通化市针对黑龙江省推送的信息，立即追踪到该个体营销人员并对其进行核酸检测，检测结果为阳性，经临床专家

作者最新文章
人民日报社论：共同奏响和平、团结、进步的时代乐章
北京2022年冬残奥会中国体育代表团成立
总书记牵挂的粮食安全丨“要实打实地调整结构”
相关文章
香港疫情不断外溢，传来频频噩耗，有人开始往中国“千里送
苏州疫情不断外溢，官方通报释放一关键信号，这件事可千万别

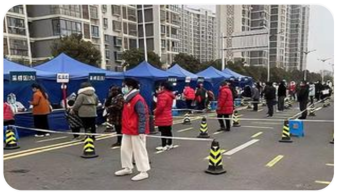

一图读懂！预防诺如病毒这样做

组会诊，诊断为无症状感染者。这名“个体营销人员”，即吉林省日后在疫情通报中反复提及的“1月13日通报的无症状感染者3”。

该名男子核酸检测阳性后，通化市对其密切接触人员开展追踪排查，在其授课的源升品质生活坊员工中，检测发现4例与其关联的无症状感染者。

在此之后，4天时间内，4例、2例、13例、13例、66例......与其关联的病例越来越多。

1月13日新增4例相关：无症状感染者3核酸检测阳性后，通化市对其密切接触人员开展追踪排查，在其授课的源升品质生活坊员工中，检测发现4例与其关联的无症状感染者。

1月14日新增2例相关：吉林省卫健委官方通报，1月13日0-24时，全省新增无症状感染者2例，均系1月13日通报的黑龙江省输入无症状感染者3的密切接触者。

1月15日新增13例相关：据通报1月14日0-24时，全省新增无症状感染者14例，其中13例均与1月13日通报的黑龙江省输入无症状感染者3有关联。

1月16日新增13例相关：据通报1月15日0-24时，全省新增无症状感染者13例，均与1月13日通报的黑龙江省输入无症状感染者3有关联。健康时报记者注意到，这些无症状感染者为1月8日在公主岭市范家屯镇艾尚瀚邦养生会馆参加该名男子的培训授课。

1月17日新增66例相关：据通报1月16日0-24时，全省新增无症状感染者63例，其中62例与1月13日通报的黑龙江省输入无症状感染者3有关联，除此之外还有4例确诊病例皆为1月10日在通化市源升品质生活坊参加1月13日通报的无症状感染者3的培训授课。

1月17日，吉林省疫情防控发布会再次通报，截至1月17日14时，吉林省累计报告34例确诊，无症状感染者80例。吉林省本次疫情为黑龙江省望奎县无症状感染者输入后引发本地传播。目前报告的114例感染者中，有9例为黑龙江省输入，105例为本地续发感染。在续发感染者中，3例为省外输入感染者的家庭成员，另102例感染者为同一输入病例林某传播。林某从事个体营销职业，黑龙江籍，近期多次往返于黑吉两省。

最初的无症状感染者已确诊

1月13日，据吉林省卫健委通报：

无症状感染者3：男，1976年出生，黑龙江省哈尔滨市呼兰区人，个体营销人员。

1月5日从黑龙江南岔乘K350次列车（11车13号）抵达哈尔滨西站，与1月11日0-8时通报的无症状感染者1和2同车厢。

1月7日从黑龙江双城堡乘Z174次列车（2车6号）抵达长春站，约15时从黄河路客运站乘客车到公主岭市范家屯镇。

1月8日约11时从公主岭市范家屯镇乘客车返回长春站，乘坐K1383次列车（2车070号）返回黑龙江双城堡。

1月9日从哈尔滨西站乘D124次列车（5车5A号）至长春站，随后乘坐地铁1号线到达

苏州至少11名感染者同日到过这家饭店

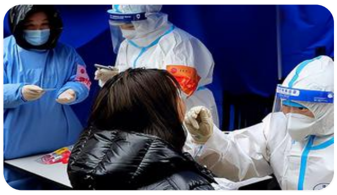

新冠疫情结束了吗？世卫组织亮明最新立场，谭德塞一句话说中

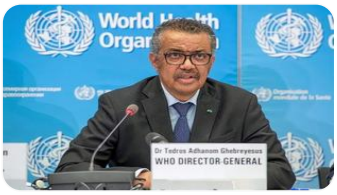

长春市高速客运站，于12时35分乘客车从长春市到通化市。

1月10日至11日在源升品质生活坊进行培训授课。

1月12日，通化市针对黑龙江省推送的该人为密切接触者的信息，立即追踪到该人并对其进行核酸检测，检测结果为阳性，经临床专家组会诊，诊断为无症状感染者。

1月16日，该无症状感染者转为确诊病例。

无症状感染者的呼吸道病毒并不一定比有症状的少

“无症状感染者的呼吸道病毒并不一定比有症状的少。”对此，北京佑安医院感染科副主任医师李侗曾表示，新冠病毒感染者的传播的能力取决于本身病毒量的多少，以及传播传染性飞沫的能力。之所以说无症状感染者传染性弱，是因为没有咳嗽咳痰这样的一些症状，从呼吸道排出病毒较少。

“整体来说，除了飞沫传播之外，还要看接触人时的空间环境情况，温度湿度对感染也都有影响”，李侗曾主任提示称，如果在相对密闭空间，例如电梯、车厢、房间内，人员聚集时，有可能传染多个人。由于此次吉林疫情的超级传播者是一位培训讲师，无症状感染者不咳嗽，但是说话时也会排出病毒。

“无症状感染者和轻症感染者其实界限容易突破，有人觉得嗓子痒轻微咳嗽就是没有症状，可能平时就这样，问起来就说没有症状，如果发现核酸阳性，再仔细问，可能就会说有咽部痒痛轻微咳嗽，就可以诊断轻症感染者了，这个其实也很常见。”李侗曾主任说道。

据美国疾控中心报告称，截至目前，全美新冠感染者有一半以上都是经过无症状感染者传播导致，可见无症状感染者的传播威力也在发生改变。

“之前的病毒跟现在的病毒有可能不一样。病毒变异有可能引起传播率的增强。”宾夕法尼亚大学医学院病理及实验医药系研究副教授张洪涛表示，一旦病毒感染人体后，每个人的症状也是各不相同，释放出来的病毒多少也不一样，这就取决于个人的免疫力。他强调，接种新冠疫苗是目前最有效的办法。接触人较多的职业应该优先接种疫苗，打疫苗的感染的可能性减少，传播的可能性就会减少。

此前，中国疾控中心副主任冯子健介绍，新冠肺炎感染者里有相当高比例的人并不传播，不会造成二代病例；但有少部分感染者或病人的传播数会超过基本传播数——3以上，会传播超过3个二代病例，这就构成了超级传播现象，这是新冠的一个非常重要的特征。他表示，新冠肺炎是超级传播现象非常突出的疾病，减少聚集性活动，是消除超级传播现象的重要措施。

(来源:健康时报)

举报/反馈

发表评论

发表神评妙论

发表

评论列表（2条）

游走aYU

东北现在串屯的小商小贩。农村集贸市场，滑雪场所，都很容易造成人员聚集.应该加以管制。让他们扫行程码健康码，医院陪护家属。应该发给发陪伴证！有利于日后排查。

2021-01-18 回复 3

为中华之崛起而喝彩

应加大力度深入研究病毒检测试剂，让病毒检测更高效，更精准。病毒检测重中之重。

2021-01-19 回复 赞

没有更多啦

设为首页 © Baidu 使用百度前必读 意见反馈 京ICP证030173号
京公网安备11000002000001号
